# Metabolic Activity and Functional Diversity Changes in Sediment Prokaryotic Communities Organically Enriched with Mussel Biodeposits

**DOI:** 10.1371/journal.pone.0123681

**Published:** 2015-04-29

**Authors:** Thomas Pollet, Olivier Cloutier, Christian Nozais, Christopher W. McKindsey, Philippe Archambault

**Affiliations:** 1 Laboratoire d’écologie benthique, Institut des sciences de la mer, Université du Québec à Rimouski, Rimouski, Québec, Canada; 2 Département de biologie, chimie et géographie, Université du Québec à Rimouski, Rimouski, Québec, Canada; 3 Ocean and Environmental Sciences Division, Maurice-Lamontagne Institute, Fisheries and Oceans Canada, Mont Joli, Québec, Canada; University of Waikato (National Institute of Water and Atmospheric Research), NEW ZEALAND

## Abstract

This experimental microcosm study reports the influence of organic enrichments by mussel biodeposits on the metabolic activity and functional diversity of benthic prokaryotic communities. The different biodeposit enrichment regimes created, which mimicked the quantity of faeces and pseudo-faeces potentially deposited below mussel farms, show a clear stimulatory effect of this organic enrichment on prokaryotic metabolic activity. This effect was detected once a certain level of biodeposition was attained with a tipping point estimated between 3.25 and 10 g day^-1^ m^-2^. Prokaryotic communities recovered their initial metabolic activity by 11 days after the cessation of biodeposit additions. However, their functional diversity remained greater than prior to the disturbance suggesting that mussel biodeposit enrichment may disturb the functioning and perhaps the role of prokaryotic communities in benthic ecosystems. This manipulative approach provided new information on the influence of mussel biodeposition on benthic prokaryotic communities and dose-response relationships and may support the development of carrying capacity models for bivalve culture.

## Introduction

As pointed out by Pearson and Rosenberg [1] four decades ago, the organic enrichment of marine waters and sediments remains one of the most universal environmental disturbances. Bivalve aquaculture is one of the fastest growing sectors in the food growing industry, with the number of farms and total production of bivalves increasing continuously over the past several decades. For example, according to the FAO (http://www.fao.org/fishery/statistics/en), the worldwide production of mussels of ~70 thousand tonnes in 1950 has since increased to ~1.8 million tonnes in 2011. However, despite the broad benefits provided by bivalve farming (particularly economic interests), bivalve culture is also known to have negative impacts on the benthic ecosystem, particularly due to the influence of biodeposition [2, 3] as it greatly influences pelagic-benthic coupling [4, 5]. Indeed, suspension-feeding bivalves have a great filtration capacity and may greatly impact plankton communities when farmed at high densities [6]. As mentioned by Navarro and Thompson [7], only a fraction of the food particles ingested by bivalves is assimilated for respiration, growth, and reproduction. The other portion, known as biodeposits, is released as faeces and undigested deposits (pseudo-faeces), both of which are organically rich and thus may lead to an accumulation of organic matter on the seafloor with consequent impacts on benthic ecosystem functioning.

The effects of mussel biodeposit accumulation on benthic ecosystems have been widely studied over the past several decades [8–15]. Most of these studies have focused on macroorganisms, including macrofaunal and meiofaunal benthic communities. As pointed out by McKindsey et al. [13], the observed level of culture-related impacts on benthic communities varies greatly between studies. While some studies have not detected impacts related to mussel culture on infaunal communities [16], others have shown that mussel biodeposition may impact the structure and composition of meiofaunal [9] and/or infaunal [14, 15, 17, 18] communities as well as nutrients fluxes [19, 20]. Some studies also suggest threshold values for organic enrichment due to bivalve biodeposition that must be surpassed for benthic communities to be impacted; for example, studies have reported this threshold to be 15 g of biodeposits m^-2^ day^-1^ [21], from 16.8 to 19 g m^-2^ day^-1^ [22], and from 4.4 to 8.8 g m^-2^ day^-1^ [15]. Although bacteria are known to be of great importance in the degradation of organic matter, and thus to play a key role in biogeochemical cycles and the functioning of aquatic food webs, such information on benthic prokaryotic communities remains scarce. Few studies have assessed how organic loading due to bivalve biodeposition impacts benthic prokaryotic communities [9, 11, 23–25]. Furthermore, although studies have shown how mussel biodeposition impacts the abundance, production, and composition of benthic prokaryotic communities, none have examined how these changes impact the metabolic activity and functional diversity of these communities. As aerobic bacteria use oxygen to degrade organic matter, great metabolic activity may generate hypoxic or anoxic conditions with consequent impacts on sediment geochemistry, meio/macrofaunal communities, and the functioning of benthic ecosystems. Thus, in the context of sustainable development, a greater understanding of the influence of mussel biodeposits on the functional activity of benthic bacteria and subsequent ecosystem modifications will better enable the mussel farming industry to expand while preserving the integrity of the ecosystem. Note that mussel biodeposit production may vary greatly with mussel species, age/size, and diet [26]. Thus the quantity of biodeposits that accumulate on bottom sediments may vary greatly over short (hourly or daily) time scales [12, 13]. In this context, it is important to consider the influence of these temporal variations in biodeposit production (increasing, decreasing or stable) on the metabolic activity of benthic prokaryotic communities.

The objectives of the present study were to determine (i) the metabolic reaction of benthic prokaryotic communities to organic loading due to mussel biodeposition, (ii) whether there is a tipping point beyond which the metabolic activity of benthic prokaryotic communities is significantly affected by biodeposit enrichment, and (iii) if prokaryotic metabolic activity and diversity recover to their initial state after several days without enrichment. This information is essential to better understand the mechanisms by which organic enrichment due to bivalve biodeposition may impact the benthic environment and is thus important in the context of ecosystem management. Our general hypothesis is that mussel biodeposits influence the metabolic activity of benthic prokaryotic communities only slightly up to a tipping point, beyond which prokaryotic functional capacity is modified significantly. We also predict that, following several days without enrichment, organic resources become limiting for benthic prokaryotic communities and thus their metabolic activity and diversity recover their initial state.

## Materials and Methods

### Sediment collection and laboratory microcosm experiment

The muddy sediment used to perform the microcosm experiment was collected during low tide in August 2013 near île Canuel (Rimouski, Quebec, Canada, 48°26'45"N 68°35'5"W) and was free from mussel biodeposits (no mussel farms were evident within several hundred kilometers). No specific permissions were required for sampling in this location and we confirm that our study did not involve endangered or protected species. Only surface sediments were collected to avoid anoxic sediments. The collected sediment was brought to the lab, sieved through a 1mm mesh to eliminate macrofauna, and gently mixed and homogenized. Microcosms consisted of 15 independent experimental units (5L, Fisherbrand histology buckets, 19.4 cm diameter) that had been washed, abundantly rinsed with deionised water and autoclaved. Each of the 15 microcosms was filled with the homogenized sediment to 2 cm depth, and covered by 2.5L of autoclaved and filtered (0.2μm) seawater. Microcosms were randomly placed in two basins connected to a constant water flow to keep a constant temperature (medium temperature ≈ 9°C throughout the experiment). Each microcosm was constantly oxygenated (air bubbler), maintained in the dark to avoid primary production, and left for six days to stabilize before starting the experiment. After this acclimatization period, the experiment started August 8, 2013 and lasted 18 days.

### Experimental design

**Fig 1:**
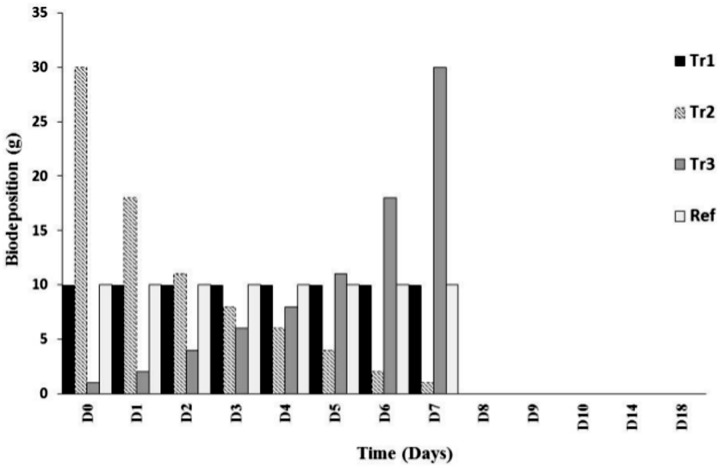
Description of the experimental design used to evaluate the influence of mussel biodeposit enrichment on benthic prokaryotic communities. Tr = Treatments. “Tr1” = constant organic matter supply (10g day^-1^ from D_0_ to D_7_). “Tr2” and “Tr3” were opposites and simulated decreasing (Tr2) and increasing (Tr3) quantities of biodeposit supply. “Ref” was similar to Tr1 except that the organic matter contained in biodeposits had been previously burned. Day 7 (D_7_) corresponds to the last day of matter addition and when all treatments had received the same quantity of matter (80g).The experiment was done over 18 days (D_0_ to D_18_). The red arrows represent the five sampling times.

Several thousand mussels were placed in large basins and biodeposits (faeces and pseudo-faeces) collected regularly and then immediately preserved at -20°C. Biodeposits were then freeze-dried to preserve organic matter (OM) quality and homogenized. During the first seven days of the experiment, different quantities of freeze-dried mussel biodeposits were added to microcosms daily (Fig 1). A total of five treatments were established in triplicate. The treatment Tr1 comprised a constant organic matter (freeze-dried mussel biodeposits) loading for the first eight days of the experiment (a rate equivalent to 10g m^-2^ d^-1^ from D_0_ to D_7_). This rate corresponds roughly to the quantity of biodeposits that accumulate on bottom sediment surfaces in a farm with a mussel density of ≈ 450 m^-2^ [12, 15]. Treatments 2 and 3 (Tr2 and Tr3) were opposites and represented decreasing (Tr2) and increasing (Tr3) quantities of biodeposit supply (Fig 1). Treatment 4 (“Ref”) was similar to Tr1 except that the organic matter contained in biodeposits had been removed by burning (see the protocol below). This treatment was done to estimate the effect of adding a volume of non-biologically active material on benthic prokaryotic communities. The “Ctrl” treatment was a procedural control that received no addition of organic matter. Day 7 (D_7_) of the experiment was the last day of biodeposit enrichment and, by that day, all treatments (except Ctrl) had received the same total quantity of matter (total quantity = 80g). Five different sampling time points (Fig 1) were chosen to reach our objectives (at days D_0_, D_2_, D_4_, D_9_ and D_18_). At each sampling time point, sediment cores were sampled using 10mL syringes. To avoid missing prokaryotic responses, we sampled on D_9_ and not the day following the last matter addition. Samplings on D_18_, 11 days after the last biodeposit addition, evaluated the ability of benthic prokaryotic communities to recover their initial metabolic activity and diversity following organic enrichment.

### Physico-chemical parameters

Biodeposit organic matter content (% OM) was estimated prior to starting the experiment as the difference between the dry weight of the freeze dried biodeposits and the residue left after combustion at 450°C for 4 h (N = 15).

The % OM of collected sediments was similarly estimated (N = 6). For each of the 15 microcosms, temperature, pH and dissolved oxygen (% DO) were assessed at the water sediment interface at four times (D_0_, D_4_, D_9_, D_18_) using a calibrated YSI multi-parameter probe.

### Prokaryotic abundances

Prokaryotic abundances were estimated by flow cytometry. For each sample, 1g of sediment was collected, fixed with formaldehyde (2% final concentration) and immediately frozen at -80°C until processed [27]. Prior to analysis, prokaryotic cells were extracted from sediments using a protocol adapted from Glud and Middelboe [28]. An initial analysis was used to estimate the minimum number of successive extractions required to obtain a good extraction efficacy and extract the maximum of prokaryotic cells by performing four successive extractions on each of three individual samples from the initially sampled sediments. Briefly, 35 mL of seawater (0.2 μm filtered) and sodium pyrophosphate (5mM final concentration) were added to the tubes with sediments, which were then vortexed for 10 seconds and sonicated for five minutes using an ultrasonic bath (VWR Symphony). Samples were then centrifuged for five minutes (3000 rpm, 20°C) and the supernatant removed and stored in a 50 ml Corning tube. Three subsequent extractions were done following the same protocol on the same sediment samples. The extracted samples were then diluted in TE 1X buffer (1:200 v/v). 1μL of fluorescent micro-beads and 0.3 μL of SYBR Green I were added to each sample as an internal standard, which was incubated for 10 minutes in the dark prior to flow cytometry analysis (cytometer Epics Altra, Beckman Coulter). Prokaryotic abundances were thus estimated for each extraction. Few differences in terms of prokaryotic cell abundances were obtained between the third and the fourth extraction (S1 Fig) and we considered that almost all bacterial cells have been extracted. An average of 96% of the total prokaryotic cells were thus obtained after three successive extractions and we considered these 96% as a good estimate of the total quantity of extractable prokaryotic cells in samples. Samples were thus subjected to three successive extractions, pooled, and then analysed using flow cytometry. Prokaryotic abundances are expressed as the number of bacteria per gram (dry weight) of sediment.

### Prokaryotic Community Level Physiological Profiling (CLPP)

We used Biolog EcoPlates to characterize the CLPP (metabolic activity and diversity) of benthic prokaryotic communities submitted to the different organic matter enrichments. Briefly, Biolog Ecoplates consist of three replicates of 31 wells containing different carbon sources and three control wells without carbon sources (http://www.biolog.com/products-static/microbial_community_overview.php). Each well also contains a minimal growth medium and tetrazolium salt which turns purple in the presence of an active electron transfer system, indicating that the substrate is being utilized by prokaryotic communities [29]. For each sample, prokaryotic cells were extracted from sediment following Gillan et al. [30]. Briefly, for each microcosm, 2g of sediment were collected and added to 10 mL of 0.2 μm filtered seawater. Samples were then sonicated for 45 seconds using an ultrasonic bath (VWR Symphony), and the suspensions centrifuged for 5 minutes at 750rpm (at 4°C) to precipitate mineral particles. Prokaryotic cell suspensions (150 μL) were placed in each well of microplates, which were then incubated at 20°C in the dark for 120h. All treatment triplicates (e.g., all Tr1 samples for a given date) were placed in the same microplate. The optical density (OD) of each well was recorded each 24h at 590nm (OD590) using a microplate spectrophotometer (BIOTEK Powerwave XS2). The metabolic activity of prokaryotic communities was characterized as the evolution of the Average Well Color Development (AWCD) during the 120h of incubation such that AWCD = Σ ODi/31 [31], where ODi is the optical density value from each well, corrected by subtracting the value observed for the control wells. In parallel, we estimated the percentage of functional diversity (%FD) as the percentage of the 31 carbon substrates assayed that were utilized (%FD = 100 * number of positive carbon source wells / 31). This value may vary between 0 to 100%, indicating low and high diversity, respectively. In this approach, we considered a carbon substrate as having been utilized when an ODi value was greater than 0.25 [32].

### Statistics

Variation among treatments was analyzed using repeated measures analysis of variance (rANOVA) as the 15 microcosms were sampled repeatedly (at D_0_, D_2_, D_4_, D_9_ and D_18_). As AWCD values were most often greatest at 120h of incubation, only these values were used in the statistical analysis. When significant differences (P<0.05) were observed, a Tukey’s pairwise comparison test was used to determine which treatments differed significantly. All statistical analyses were performed using Past software (http://folk.uio.no/ohammer/past/).

## Results

### Physico-chemical parameters

At the beginning of the experiment, sediment and biodeposits contained an average of 9.1 ± 2.8% and 15.3 ± 0.3% of organic matter, respectively. Table 1 shows the temperature, pH, and dissolved oxygen (% DO) values estimated for each treatment at four sampling time points (D_0_, D_4_, D_9_, D_18_). No physico-chemical parameters differed significantly (P>0.05) among treatments at any of the times sampled.

**Table 1:** Physico-chemical parameters.

	D_0_			D_4_			D_9_			D_18_		
	Temp	pH	DO	Temp	pH	DO	Temp	pH	DO	Temp	pH	DO
**Ctrl**	9.9 ± 0.07	7.93 ± 0.01	81.9 ± 0.5	8.7 ± 0.04	7.97 ± 0.01	85.7 ± 0.8	8.8 ± 0.04	8.06 ± 0.01	92.9 ± 0.5	8.6 ± 0.06	8.18 ± 0.04	107.0 ± 0.7
**Tr1**	9.9 ± 0.12	7.92 ± 0.06	86.2 ± 5.1	8.7 ± 0.07	7.93 ± 0.03	83.3 ± 2.7	8.8 ± 0.05	8.04 ± 0.01	92.4 ± 0.6	8.6 ± 0.12	8.19 ± 0.03	105.8 ± 1.1
**Tr2**	9.9 ± 0.06	7.95 ± 0.01	82.7 ± 0.4	8.8 ± 0.05	7.96 ± 0.02	85.7 ± 0.4	8.9 ± 0.04	8.06 ± 0.01	93.0 ± 0.7	8.6 ± 0.01	8.20 ± 0.03	107.2 ± 1.0
**Tr3**	9.9 ± 0.06	7.96 ± 0.02	82.6 ± 0.3	8.8 ± 0.01	7.96 ± 0.01	85.8 ± 0.6	8.9 ± 0.04	8.03 ± 0.02	93.3 ± 0.3	8.5 ± 0.01	8.19 ± 0.01	106.2 ± 1.7
**Ref**	9.8 ± 0.10	7.94 ± 0.01	82.1 ± 0.1	8.7 ± 0.12	7.97 ± 0.02	85.4 ± 0.8	8.8 ± 0.12	8.04 ± 0.01	93.1 ± 0.4	8.5 ± 0.10	8.18 ± 0.03	106.4 ± 0.8

Variation in temperature (Temp, in °C), pH and Dissolved Oxygen (DO, in percentage) observed in microcosms at four sampling time points (D_0_, D_4_, D_9_ and D_18_) for the 5 treatments. N = 3 (Mean ± SD).

### Prokaryotic abundances

Variation in prokaryotic abundances during the microcosm experiment is shown in Table 2. Average prokaryotic abundances ranged from 2.3 ± 0.6 to 3.3 ± 1.3 x 10^9^, 1.6 ± 0.2 to 2.4 ± 1.2 x 10^9^, 1.6 ± 0.3 to 2.7 ± 0.8 x 10^9^, 1.3 ± 0.4 to 2.5 ± 0.6 x 10^9^ and 1.3 ± 0.3 to 2.4 ± 0.7 x 10^9^ per gram of sediment on days D_0_, D_2_, D_4,_ D_9_ and D_18_, respectively. Variation in values between treatments and controls did not differ significantly (P>0.05) on any sampling date.

**Table 2:** Prokaryotic abundances estimated by flow cytometry.

	Ctrl	Tr1	Tr2	Tr3	Ref
**D_0_**	3.1 ± 0.2	2.3 ± 0.6	3.3 ± 1.3	2.3 ± 1.1	3.1 ± 1.4
**D_2_**	1.9 ± 0.3	2.4 ± 1.2	2.2 ± 0.3	2.1 ± 0.6	1.6 ± 0.2
**D_4_**	2.6 ± 0.6	1.8 ± 0.7	2.0 ± 0.1	2.1 ± 0.7	1.6 ± 0.3
**D_9_**	2.5 ± 0.4	1.4 ± 0.2	2.3 ± 0.9	1.3 ± 0.4	1.5 ± 0.3
**D_18_**	1.7 ± 0.6	2.4 ± 0.6	1.3 ± 0.3	1.4 ± 0.6	1.6 ± 0.2

Mean prokaryotic abundances (×10^9^ per gram of sediment) estimated at each sampling time point (D_0_, D_2_, D_4_, D_9_ and D_18_) in the different treatments. N = 3 (Mean ± SD).

### Prokaryotic metabolic activity and diversity

**Fig 2:**
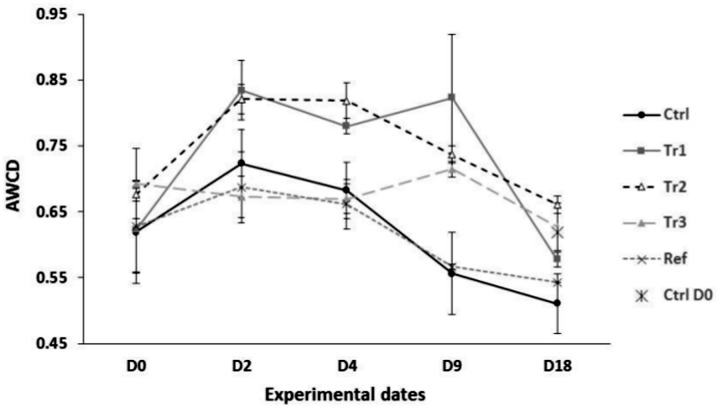
Assessment of the Average Well Color Development (AWCD) for all treatments. Mean (± SE) AWCD values measured at 120 h incubation for microcosms subjected to various treatments (see text for details).

**Fig 3:**
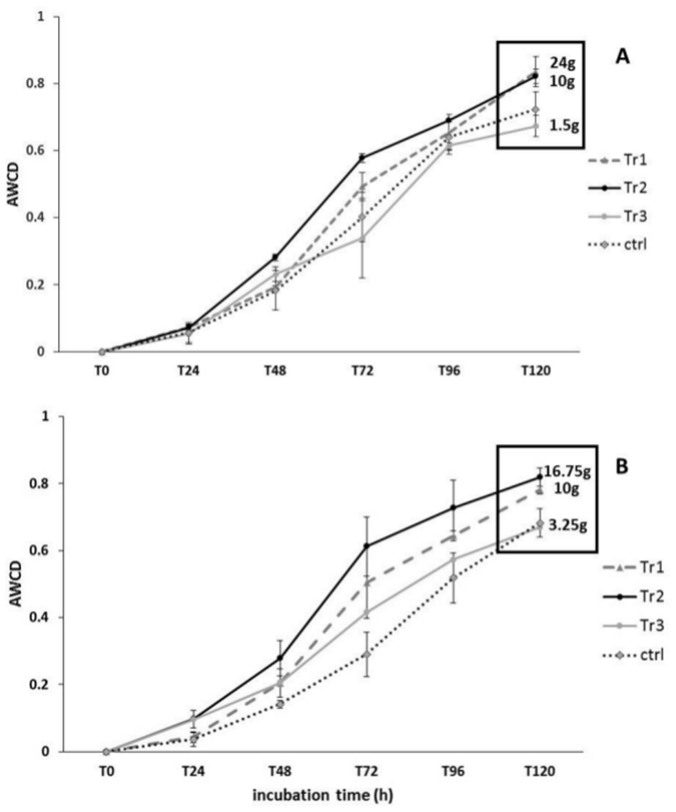
Variation in AWCD at D_2_ and D_4_. Variation in AWCD values (Mean ± SE) for the treatments Tr1, Tr2, Tr3 and the control (ctrl) at D_2_ (A) and D_4_ (B). For each treatment, the mean quantity (g) of mussel biodeposits daily received by prokaryotic communities at these two sampling time points is indicated.

Fig 2 shows the AWCD for all treatments at the beginning of the experiment and prior to adding biodeposits (D_0_), at D_2_, and D_4_, corresponding to when treatments had received different total quantities of biodeposits, when all treatments (except the control) had received the same quantity of biodeposits (80g, D_9_) and one week thereafter, at the end of the experiment (D_18_). At D_0_, AWCD did not differ among treatments. At D_2_ and D_4_, AWCD values were significantly greater in Tr1 and Tr2 than in Tr3, Ctrl and Ref treatments, the latter which did not differ. At D_9_ (Fig 2), AWCD was significantly higher in all microcosms that had received organic matter (Tr1, Tr2 and Tr3) than in Ctrl and Ref microcosms, which did not differ. At D_18_, AWCD did not differ among treatments or from the AWCD values observed at D_0_. These results seem to indicate that a threshold deposition rate is required to shift prokaryotic metabolic activity. This threshold was reached by D_2_ in both the Tr1 and Tr2 treatments, which both showed increased (relative to Ref and Ctrl treatments) metabolic activity on both D_2_ and D_4_ and on D_9_. Biodepostion rates to Tr1 and Tr2 microcosms averaged 10 and 24 g day^-1^ (D_2_) and 10 and 16.75 g day^-1^ (D_4_), respectively (Fig 3A and 3B). In contrast, the Tr3 treatment had been subjected to an average biodeposition rate of only 1.5 and 3.25 g day^-1^ by D_2_ and D_4_, respectively, and only showed increased metabolic activity (relative to Ctrl and Ref treatments) by D_9_, when the average biodepostion rate over the experiment in Tr1, Tr2, and Tr3 reached 10 g day^-1^ and all three treatments had increased metabolic activity. It is not clear exactly where the threshold is, but given the above, it appears to be between 3.25 and 10 g day^-1^.

**Fig 4:**
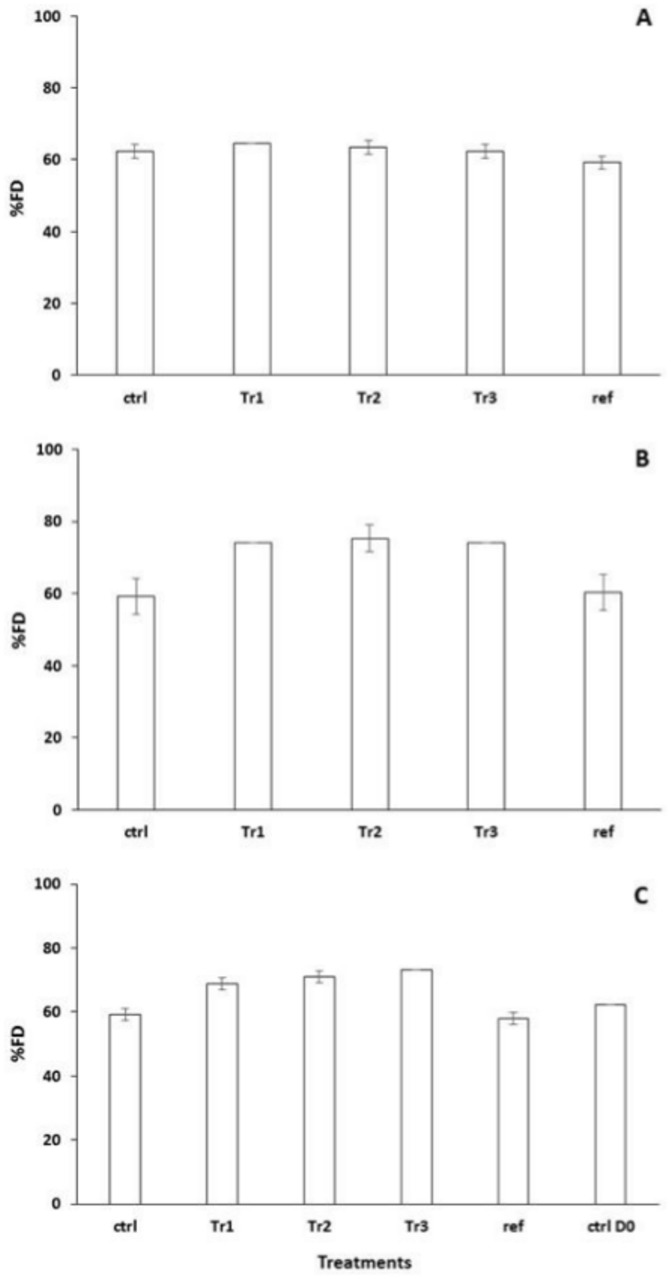
Assessment of the prokaryotic functional diversity (%FD). Variations (Mean ± SE) in %FD at D_0_ (A), D_9_ (B) and D_18_ (C) in the different treatments.

On D_0_, %FD did not differ among treatments (Fig 4A). In contrast, %FD was greater in Tr1 and Tr2 microcosms than in microcosms for the other treatments (which did not differ) on D_2_ and D_4_, and on D_9_ and D_18_ was significantly greater in all microcosms that had received biodeposits (Tr1, Tr2 and Tr3) than in the Ctrl and Ref microcosms (Fig 4B and 4C). Note that the greatest differences in %FD between microcosms supplemented with biodeposits and the Ctrl and Ref microcosms were related to carbohydrate utilization. While alpha D lactose and beta methyl D glucoside were highly utilized in all treatments at D_0_, only the treatments that received subsequent additions of biodeposits (Tr1, Tr2 and Tr3) continued to metabolize these organic substrates at D_9_ and D_18_. In addition, while D xylose was not utilized at the beginning of the experiment (D_0_) in any of the treatments, it too was highly degraded in the organically enriched Tr1, Tr2 and Tr3 microcosms but not in the Ctrl and Ref microcosms at D_9_ and D_18_. These results provide further support for the notion that the tipping point for prokaryotic communities is somewhere between 3.25 and 10 g biodeposits m^-2^ d^-1^.

## Discussion

To the best of our knowledge, this is the first study to examine the impact of mussel biodeposition on the metabolic activity and functional diversity of benthic prokaryotic communities. Prokaryotic abundances in the experimental microcosms were very similar to those commonly reported in coastal marine sediments [11, 33–35]. Mussel biodeposit enrichment did not seem to impact prokaryotic abundances as similar values were observed in all microcosm treatments at different sampling times. Previous studies on the impact of this type of organic matter enrichment have found contrasting results. Although Danovaro et al. [11] also observed little effect of mussel farming on prokaryotic abundances, other studies have shown prokaryotic densities to increase with mussel biodeposit enrichment [9, 24]. Unlike the experimental microcosm approach employed in the current study, previous studies [9, 11] were done *in situ* under natural conditions by comparing communities from locations below mussel farms to others some distance from farms. Any effects observed are in some measure confounded as sites were necessarily not exposed to the same environmental conditions, potentially accentuating or reducing the effects of mussel biodeposits *per se* on these communities. For example, effects observed by Mirto et al. [9] may have been due to differences in light among locations as one of the main effects observed was a proliferation of cyanobacteria. Similarly, whereas bivalve farm sites are often located in shallow, low-energy coastal sites [12], hydrodynamic processes (resuspension and advection) may have accounted for a lack of effect of biodeposition on prokaryotic density in the more off-shore mussel farm site in the Mediterranean Sea studied by Danovaro et al. [11]. In the present study, the lack of effect of mussel biodeposit enrichment on prokaryotic abundances was not linked to other environmental variables, such as temperature, pH, dissolved oxygen, light and hydrodynamic processes, which were similar in all microcosms throughout the experiment. The only difference between biodeposit treatments and control and reference treatments was the enrichment with mussel biodeposits. The observed lack of effect on prokaryotic abundances may be due to a strategic choice of bacteria to favour cell growth rather than the cell division. This hypothesis is supported by work by Lavrentyev et al. [36], who observed increased prokaryotic size structure in treatments with mussels, and with work by Danovaro et al. [11] that showed that cell division rates did not differ between sites impacted by mussel biodeposition and reference sites. However, prokaryotic abundance is a function of prokaryotic growth rate and may also be regulated by viral lysis and predators in impacted sediments. This latter mechanism may be less likely as Lavrentyev et al. [36] described a decrease in the ratio of protozoan to prokaryotic biomass in mussel treatments, suggesting a lower degree of bacteriovory in these treatments. However, Fabiano et al. [37] observed a rapid proliferation of flagellates and ciliates in mussel biodeposit-impacted seawater, suggesting a potential increase in grazing activity. The influence of predators on prokaryotic communities submitted to mussel biodeposits thus remains unclear and may be examined in future studies.

Contrasting results are also observed in the literature concerning the influence of mussel biodeposits on benthic prokaryotic activity. Although Danovaro et al. [11] observed no differences between the growth rates of bacterial communities below mussel lines and those in a reference area, Grenz et al. [23] showed that bacterial production and enzymatic activity increase due to mussel biodeposition. The results of our study, done under controlled conditions, showed increased metabolic activity of benthic prokaryotic communities affected by mussel biodeposits. As discussed above, environmental conditions were very similar between the different treatments, suggesting that the observed increase in prokaryotic metabolic activity in the present experiment was due to mussel biodeposition.

Our experimental approach allows us to further examine the influence of mussel biodeposits on benthic prokaryotic communities to identify a tipping point beyond which the rate of mussel biodeposition significantly influences the metabolic activity and functional diversity of prokaryotes. Our results suggest that this threshold is between 3.25 and 10 g of mussel biodeposits m^-2^ day^-1^(Fig 3). Based on Callier et al. [38], this quantity of biodeposits corresponds to a density of 150 and 450 mussels m^-2^. The level of biodeposition at which we observed a tipping point is quite similar to other estimates of tipping points based on studies on benthic infaunal communities [12, 21, 22, 38]. The tipping point proposed here is particularly close to the 4.4–8.8 g m^-2^ day^-1^ estimated by Robert et al. [15], based on benthic macrofaunal communities. The existence of this tipping point may explain the lack of bacterial response (in terms of growth rate) in the study by Danovaro et al. [11], who found that benthic bacterial metabolism under a mussel farm did not differ from that in sediments that were not subjected to mussel biodeposition. This may have been due to hydrodynamic conditions that dispersed bivalve biodeposits and thus decreased the quantity of organic matter that was available for prokaryotic degradation [11]. Indeed, that study found that organic fluxes to the bottom were in the range of 0.5–1.15 g organic matter m^-2^ d^-1^ in the farm and control sites. The results reported here provide a better understanding of the potential mechanisms by which organic loading due to bivalve biodeposition may impact the benthic environment and thus are important in the context of ecosystem management [39]. As bacteria use oxygen to mineralize organic matter, an increase in their metabolic capacity, as observed in the present study, could rapidly increase benthic oxygen demand, potentially leading to hypoxic or anoxic benthic conditions. Indeed, the temporal pattern in loading rates used in the present study and the reaction of the prokaryotic communities to it correspond roughly to the classification of organic enrichment for marine sediments outlined by Hargrave et al. [21]. According to this model, the lower daily loading rates used in the present study should result in oxic conditions whereas loading rates approaching 10 g m^-2^ d^-1^ should lead to polluted (Hypoxic A) conditions. The precise location of this tipping point is thus important in an ecosystem management context and should be examined in further detail in the future by estimating the daily microbial consumption of organic matter under different environmental conditions. It would also be of interest to determine the minimum quantity of mussel biodeposition required to impact prokaryotic functions and whether the intensity and/or the frequency of mussel biodeposit enrichment must be taken into account. Future studies will assess the influence of biodeposit loading regimes (e.g. chronic and sporadic) on the metabolic activity of benthic prokaryotic communities. Finally, note that we removed the macrofauna from the sediments placed in microcosms, which are also known to consume of biodeposits [10, 22]. It would be interesting in future studies to consider and estimate the consumption of biodeposits by both microbes and macrofauna to suggest a more global tipping point beyond which biodeposit-degrading benthic communities may be affected by mussel biodeposit enrichment.

The present study also shows for the first time that, beyond the tipping point, the addition of organic matter also increases the functional diversity of benthic prokaryotic communities, as benthic bacteria subjected to biodeposit additions were able to utilize a greater quantity and diversity of carbohydrates as carbon sources. In addition, prokaryotic communities subjected to biodeposit additions developed the capacity to degrade specific carbohydrates, such as the D-xylose. This suggests that mussel biodeposition may favour the expression of specific functions and modify the functional role of prokaryotic communities in surface sediments. Despite the lack of data on the benthic prokaryotic community composition in the present study and in the literature in general, these functional changes could suggest modifications in prokaryotic community composition with the development of bacteria that are able to degrade this type of carbohydrate-rich organic matter. This would be in agreement with work by Danovaro et al. [11] who showed that mussel biodeposition modifies prokaryotic community structure. Some studies [23, 37] also suggest that a significant fraction of mussel biodeposits is degraded by mussel intestinal prokaryotic communities, particularly in the first few days following biodeposition. Grenz et al. [23] suggested that mussel gut-related bacteria were present in biodeposits and likely important in the degradation of this organic matter. Similarly, Fabiano et al. [37] showed that biodeposits contain a rich prokaryotic fauna upon egestion, as is common for marine invertebrates [40], and that these communities were active, reaching maximum density 50 h post-egestion. We suggest that this may not have been the case in the present study due to the freeze drying step in our experimental protocol. Indeed, this step is known to negatively affect both the viability and physiological state of bacteria [41, 42], particularly when microbes are not protected by protective agents that are indubitably important for the survival of prokaryotic cells. Thus, this procedure likely removed the majority of mussel gut-related micro-organisms. Nonetheless, some prokaryotes may have survived freeze drying and were introduced into the microcosms with biodeposits. However, had this been the case, it would be expected that this would have immediately stimulated prokaryotic activity and functional diversity, which did not seem to be the case as the Tr3 microcosms only reacted after 9 days of receiving biodeposits. Moreover, the capacity to utilize specific carbon sources persisted even after 11 days without additional biodeposit supply, suggesting that changes in functional diversity were probably not related to intestinal prokaryotic communities which would, *a priori*, only be active in the first few days following biodeposit additions [23, 37]. Further studies addressing the influence of mussel biodeposits on prokaryotic community composition need to be done in the future to address this knowledge gap.

In the context of sustainable development, it is important to determine if benthic communities that have been affected by biodeposit-related enrichment may recover to their initial state in terms of functional activity and diversity. Little information concerning such post-disturbance responses is currently available [23]. The present study shows that the metabolic activity of prokaryotic communities recovered to the initial state within eleven days following the last biodeposit additions. AWCD values of all treatments at day D_18_ were similar to those recorded at day D_0_ (Fig 2). These observations are in agreement with those of Grenz et al. [23], who showed that bacterial production returned to background levels eight days following mussel biodeposit enrichment, suggesting that the labile fraction of organic matter in biodeposits may have been completely consumed within this time period and become limiting for bacterial communities. These authors also suggest that this organic matter may be rapidly (several days) degraded and thus have a temporally limited impact on benthic prokaryotic communities. This timing is also supported by work of Giles and Pildich [43], who added mussel biodeposits to benthic sediments in the lab and found that they were degraded at a rate of about 0.16 day^-1^, for a half-life of about 4.3 days. Although prokaryotic metabolic activity declined rapidly following the cessation of biodeposit additions, the expression of specific functions related to mussel biodeposit additions persisted. This suggests that various and higher functional capacities are conserved, at least in the short term, following mussel biodeposit enrichment and may continue to impact the functioning and perhaps the role of prokaryotic communities in benthic ecosystems. The next logical step would be to examine if these changes in prokaryotic functional diversity are persistent or temporary.

In conclusion, the present manipulative experiment showed a clear influence of mussel biodeposit enrichment on prokaryotic functional activity and diversity. This effect was evident only above a certain rate of biodeposit supply with a tipping point estimated between 3.25 and 10 g m^-2^ day^-1^. Although the studied prokaryotic communities recovered their initial metabolic activity within 11 days following the cessation of biodeposit additions, the functional diversity (number of carbon sources they are able to utilize) of the communities remained greater than prior to the disturbance. These results are clearly useful for ecosystem management and may be incorporated into models to favour the sustainable development of bivalve culture.

## Supporting Information

S1 Fig Number of bacteria obtained from each of four successive extractions. Samples 1, 2 and 3 represent three replicates of the initial sampled sediment.
(TIFF)

## References

[1] Pearson TH, Rosenberg R. Macrobenthic succession in relation to organic enrichment and pollution of the marine environment. Oceanogr Mar Biol Annu Rev. 1978; 16: 229–311.

[2] Gilbert F, Souchu P, Bianchi M, Bonin P. Influence of shellfish farming activities on nitrification, nitrate reduction to ammonium and denitrification at the water–sediment interface of the Thau lagoon, France. Mar Ecol Prog Ser. 1997; 151: 143–153.

[3] Mirto S, Fabiano M, Danovaro R, Manganaro A, Mazzola A. Use of meiofauna for detecting fish farming disturbance in coastal sediments: preliminary results. Biol Mar Medit. 1999; 6: 331–334.

[4] Zhou Y, Yang H, Zhang T, Liu S, Zhang S, Liu Q, et al Influence of filtering and biodeposition by the cultured scallop *Chlamys farreri* on benthic-pelagic coupling in a eutrophic bay in China. Mar Ecol Prog Ser. 2006; 317: 127–141.

[5] Callier MD, Weise AM, McKindsey CW, Desrosiers G. Sedimentation rates in a suspended mussel farm (Great-Entry Lagoon, Canada): biodeposition production and dispersion. Mar Ecol Prog Ser. 2006; 322: 129–141.

[6] Newell RIE. Ecosystem influences of natural and cultivated populations of suspension-feeding bivalve molluscs: a review. J Shellfish Res. 2004; 23: 51–61.

[7] Navarro JM, Thompson RJ. Biodeposition by the horse mussel Modiolus modiolus (Dillwyn) during the Spring diatom bloom. J Exp Mar Biol Ecol. 1997; 209: 1–13.

[8] Kaiser MJ, Laing I, Utting SD, Burnell GM. Environmental impacts of bivalve mariculture. J Shellfish Res. 1998; 17: 59–66.

[9] Mirto S, La Rosa T, Danovaro R, Mazzola A. Microbial and meiofaunal response to intensive mussel-farm biodeposition in coastal sediments of the western Mediterranean. Mar Pollut Bull. 2000; 40: 244–252.

[10] Christensen PB, Glud RN, Dalsgaard T, Gillespie P. Impacts of longline mussel farming on oxygen and nitrogen dynamics and biological communities of coastal sediments. Aquaculture. 2003; 218: 567–588.

[11] Danovaro R, Gambi C, Luna GM, Mirto S. Sustainable impact of mussel farming in the Adriatic Sea (Mediterranean Sea): evidence from biochemical, microbial and meiofaunal indicators. Mar Pollut Bull. 2004; 49: 325–333. 15341827 10.1016/j.marpolbul.2004.02.038

[12] Weise AM, Cromey CJ, Callier MD, Archambault P, Chamberlain J, McKindsey CW. Shellfish-DEPOMOD: modelling the biodeposition from suspended shellfish aquaculture and assessing benthic effects. Aquaculture. 2009; 288: 239–253.

[13] McKindsey CW, Archambault P, Callier MD, Olivier F. Influence of suspended and off-bottom mussel culture on the sea bottom and benthic habitats: a review. Can J Zool. 2011; 89: 622–646.

[14] Grant C, Archambault P, Olivier F, McKindsey CW. Influence of bouchot mussel culture on the benthic environment in a dynamic intertidal system. Aquacult Environ Interact. 2012; 2: 117–131.

[15] Robert P, McKindsey CW, Chaillou G, Archambault P. Dose-dependent response of a benthic system to biodeposition from suspended blue mussel (*Mytilus edulis*) culture. Mar Pollut Bull. 2013; 66: 92–104. 10.1016/j.marpolbul.2012.11.003 23219398

[16] Crawford C, Macleod CKA, Mitchell IM. Effects of shellfish farming on the benthic environment. Aquaculture. 2003; 224: 117–140.

[17] Hartstein ND, Rowden AA. Effect of biodeposits from mussel culture on macroinvertebrate assemblages at sites of different hydrodynamic regime. Mar Environ Res. 2004; 57: 339–357. 14967518 10.1016/j.marenvres.2003.11.003

[18] Callier M, McKindsey CW, Desrosiers G. Evaluation of indicators used to detect mussel farm influence on the benthos: two case studies in the Magdalen Islands, Eastern Canada. Aquaculture. 2008; 278: 77–88.

[19] Richard M, Archambault P, Thouzeau G, Desrosiers G. Influence of suspended mussel lines on the biogeochemical fluxes in adjacent water in the Îles-de-la-Madeleine (Quebec, Canada). Can J Fish Aquat Sci. 2006; 63: 1198–1213.

[20] Richard M, Archambault P, Thouzeau G, McKindsey CW, Desrosiers G. Influence of suspended scallop cages and mussel lines on pelagic and benthic biogeochemical fluxes in Havre-aux-Maisons Lagoon, Îles-de-la-Madeleine (Quebec, Canada). Can J Fish Aquat Sci. 2007; 64: 1491–1505.

[21] Hargrave BT, Holmer M, Newcombe CP. Towards a classification of organic enrichment in marine sediments based on biogeochemical indicators. Mar Pollut Bull. 2008; 56: 810–824. 10.1016/j.marpolbul.2008.02.006 18343458

[22] Callier MD, McKindsey CW, Desrosiers G. Multi-scale spatial variations in benthic sediment geochemistry and macrofaunal communities under a suspended mussel culture. Mar Ecol Prog Ser. 2007; 348: 103–115.

[23] Grenz C, Hermin MN, Baudinet D, Daumas R. In situ biochemical and bacterial variation of sediments enriched with mussel biodeposits. Hydrobiologia. 1990; 207: 153–160.

[24] La Rosa T, Mirto S, Mazzola A, Danovaro R. Differential responses of benthic microbes and meiofauna to fish-farm disturbance in coastal sediments. Environ Pollut. 2001; 112: 427–434. 11291449 10.1016/s0269-7491(00)00141-x

[25] Danovaro R, Corinaldesi C, La Rosa T, Luna GM, Mazzola A, Mirto S et al Aquaculture impact on benthic microbes and organic matter cycling in coastal Mediterranean sediments: a synthesis. Chemist Ecol. 2003; 19: 59–65.

[26] McKindsey CW, Lecuona M, Huot M, Weise AM. Biodeposit production and benthic loading by farmed mussels and associated tunicate epifauna in Prince Edward Island. Aquaculture. 2009; 295: 44–51.

[27] Piot A, Nozais C, Archambault P. Meiofauna affect the macrobenthic biodiversity-ecosystem functioning relationship. Oikos. 2014; 123: 203–213.

[28] Glud RN, Middelboe M. Virus and bacteria dynamics of a coastal sediment: implication for benthic carbon cycling. Limnol Oceanogr. 2004; 49: 2073–2081.

[29] Garland JL, Mills AL. Classification and characterization of heterotrophic microbial communities on the basis of patterns of community level sole carbon source utilization. Appl Environ Microbiol. 1991; 57: 2351–2359. 16348543 10.1128/aem.57.8.2351-2359.1991 PMC183575

[30] Gillan DC, Pede A, Sabbe K, Gao Y, Leermakers M, Baeyens W, et al Effect of bacterial mineralization of phytoplankton-derived phytodetritus on the release of arsenic, cobalt and manganese from muddy sediments in the Southern North Sea. A microcosm study. Sci Tot Environ. 2012; 419: 98–108. 10.1016/j.scitotenv.2011.12.034 22281039

[31] Gomez E, Garland J, Conti M. Reproducibility in the response of soil bacterial community-level physiological profiles from a land use intensification gradient. Appl Soil Ecol. 2004; 26: 21.

[32] Garland JL. Analysis and interpretation of community level physiological profiles in microbial ecology. FEMS Microbiol Ecol. 1997; 24: 289–300.

[33] Kuwae T, Hosokawa Y. Determination of abundance and biovolume of bacteria in sediments by dual staining with 4, 6-diamidino-2-phenylindole and acridine orange: relationships to dispersion treatment and sediment characteristics. Appl Environ Microbiol. 1999; 65: 3407–3412. 10427027 10.1128/aem.65.8.3407-3412.1999 PMC91512

[34] Sestanovic S, Solic M, Krstulovic N, Bogner D. Volume, abundance and biomass of sediment bacteria in the eastern mid Adriatic Sea. Acta Adriat. 2005; 46: 177–191.

[35] Luna GM, Corinaldesi C, Dell’Anno A, Pusceddu A, Danovaro R. Impact of aquaculture in benthic virus-prokaryote interactions in the Mediterranean Sea. Water Res. 2013; 47: 1156–1168. 10.1016/j.watres.2012.11.036 23276430

[36] Lavrentyev PJ, Gardner WS, Yang L. Effects of the zebra mussel on nitrogen dynamics and the microbial community at the sediment-water interface. Aquat Microb Ecol. 2000; 21: 187–194.

[37] Fabiano M, Danovaro R, Olivari E, Misic C. Decomposition of faecal matter and somatic tissue of *Mytilus galloprovincialis*: changes in organic matter composition and microbial succession. Mar Biol. 1994; 119: 375–384.

[38] Callier M, Richard M, McKindsey CW, Archambault P, Desrosiers G. Responses of benthic macrofauna and biogeochemical fluxes to various levels of mussel biodeposition: an in situ ‘‘benthocosm” experiment. Mar Pollut Bull. 2009; 58: 1544–1553. 10.1016/j.marpolbul.2009.05.010 19541330

[39] McKindsey CW, Thetmeyer H, Landry T, Silvert W. Review of recent carrying capacity models for bivalve culture and recommendations for research and management. Aquaculture. 2006; 261: 451–462.

[40] Harris JM. The presence, nature, and role of gut microflora in aquatic invertebrates—a synthesis. Microb Ecol. 1993; 25: 195–231. 10.1007/BF00171889 24189919

[41] Brashears MM, Gilliland SE. Survival during frozen and subsequent refrigerated storage of *Lactobacillus acidophilus* cells as influenced by the growth phase. J Dairy Sci. 1995; 78: 2326–2335. 8747323 10.3168/jds.S0022-0302(95)76859-X

[42] Foschino R, Fiori E, Galli A. Survival and residual activity of *Lactobacillus acidophiluss* frozen cultures under different conditions. J Dairy Res. 1996; 63: 295–303.

[43] Giles H, Pilditch CA. Effects of diet on sinking rates and erosion thresholds of mussel *Perna canaliculus* biodeposits. Mar Ecol Prog Ser. 2004; 282: 205–219.

