# Supplementary figures and images for: Metabolic Activity and Functional Diversity Changes in Sediment Prokaryotic Communities Organically Enriched with Mussel Biodeposits
Source: PLoS One. 2015 Apr 29;10(4):e0123681. doi: 10.1371/journal.pone.0123681 (PMC4414560; doi:10.1371/journal.pone.0123681)

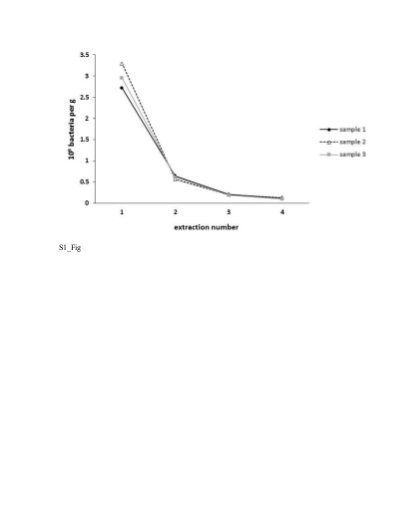

Supplement: S1 Fig — Samples 1, 2 and 3 represent three replicates of the initial sampled sediment. (TIFF) [file pone.0123681.s001.tiff]
